# SnRNA-Seq of Pancreas Revealed the Dysfunction of Endocrine and Exocrine Cells in Transgenic Pigs with Prediabetes

**DOI:** 10.3390/ijms24097701

**Published:** 2023-04-22

**Authors:** Huanqi Peng, Kaiyi Zhang, Jiakun Miao, Yu Yang, Shuang Xu, Tianwen Wu, Cong Tao, Yanfang Wang, Shulin Yang

**Affiliations:** State Key Laboratory of Animal Nutrition, Ministry of Agriculture Key Laboratory of Animal Genetics Breeding and Reproduction, Institute of Animal Science, Chinese Academy of Agricultural Sciences, Beijing 100193, China

**Keywords:** pancreas, single-nucleus RNA-sequencing, transgenic pig, TXNIP, prediabetes

## Abstract

Diabetes poses a significant threat to human health. Exocrine pancreatic dysfunction is related to diabetes, but the exact mechanism is not fully understood. This study aimed to describe the pathological phenotype and pathological mechanisms of the pancreas of transgenic pigs (PIGinH11) that was constructed in our laboratory and to compare it with humans. We established diabetes-susceptible transgenic pigs and subjected them to high-fat and high-sucrose dietary interventions. The damage to the pancreatic endocrine and exocrine was evaluated using histopathology and the involved molecular mechanisms were analyzed using single-nucleus RNA-sequencing (SnRNA-seq). Compared to wild-type (WT) pigs, PIGinH11 pigs showed similar pathological manifestations to type 2 diabetes patients, such as insulin deficiency, fatty deposition, inflammatory infiltration, fibrosis tissue necrosis, double positive cells, endoplasmic reticulum (ER) and mitochondria damage. SnRNA-seq analysis revealed 16 clusters and cell-type-specific gene expression characterization in the pig pancreas. Notably, clusters of Ainar-M and Endocrine-U were observed at the intermediate state between the exocrine and endocrine pancreas. Beta cells of the PIGinH11 group demonstrated the dysfunction with insulin produced and secret decreased and ER stress. Moreover, like clinic patients, acinar cells expressed fewer digestive enzymes and showed organelle damage. We hypothesize that TXNIP that is upregulated by high glucose might play an important role in the dysfunction of endocrine to exocrine cells in PIGinH11 pigs.

## 1. Introduction

Diabetes and its complications are a significant threat to human health. In 2021, 536.6 million people (age from 20 to 79) were estimated to have diabetes, which is predicted to increase to 783.2 million by 2045; of these, more than 90% are expected to have type 2 diabetes mellitus (T2DM) [1,2]. Beta cell dysfunction is known to cause diseases related to T2DM [1,3]. Clinically, many people with diabetes have exocrine pancreatic dysfunction (EPD). EPD is reported in 40% of patients with type 1 diabetes mellitus (T1DM) and 27% of patients with T2DM [4,5]. Given this high prevalence, more research is necessary to prove EPD’s pathogenesis and its association with diabetes. The pancreas is a bifunctional organ with internal heterogeneity. The predominant exocrine pancreas includes acinar and ductal cells, which produce and deliver digestive enzymes to the intestine. Interspersed with the exocrine pancreas are the islets of Langerhans, consisting of five different endocrine cell types, including alpha cells, beta cells, gamma cells, delta cells, and epsilon cells. It is critical to study cell-type-specific gene expression in health and disease in order to understand the molecular mechanisms involved in metabolic disorder-related diseases and those that govern pancreatic function. Previous single-cell RNA-sequencing (ScRNA-seq) studies investigating the heterogeneity of human pancreatic tissue have been mostly limited to pancreatic islets [6,7,8,9,10], and only a small number of exocrine cells from single-cell studies have been reported [11,12]. Moreover, there is no research to describe the characterization of the pancreas that considers both the exocrine and endocrine pancreas in diabetes. Nowadays, pre-clinical research on the pancreas mainly depends on animal models because there is limited access to pancreatic tissue from human patients. Rodent models are the most widely used animal models for metabolic disorder-related diseases [13]. Pigs have unique advantages in terms of the study of these diseases related to pancreatic function. For example, the anatomy and physiology of pigs are more similar to humans, porcine islets are a potential source of islet xenotransplantation, and the ethical issues related to animal research in pigs are lesser than in non-human primates [14,15].

We have successfully constructed a metabolic disorder pig model (PIGinH11) by knocking in three human disease susceptibility genes (GIPR^dn^, hIAPP, and PNPLA3^I148M^), governed by a tissue-specific expressing promoter of a beta cell or liver, to the H11 locus on chromosome 14 of the pig genome. The dominant-negative glucose-dependent insulinotropic polypeptide receptor (GIPR^dn^) inhibits islet beta cell differentiation and proliferation. The human islet amyloid polypeptide (hIAPP) promotes the apoptosis of beta cells. The patatin-like phospholipase domain-containing three variant I148M (PNPLA3^I148M^) promotes lipid deposition and increases the recruitment of inflammatory cells in the liver. Our previous studies have shown that PIGinH11 pigs exhibit impaired glucose tolerance, pancreatic fat infiltration, compensatory islet proliferation, and inflammation infiltration in the liver and visceral adipose tissue after 12 weeks of high-fat and high-sucrose diet (HFHSD) induction. They showed the typical characteristics of glucolipid metabolism disorders found in human patients [16]. In this study, we performed a prolonged HFHSD induction in transgenic and wild-type pigs. For pancreatic tissues, pathological features were evaluated in three dimensions: serological indices, pathological sections, and electron microscopy. Moreover, to obtain comprehensive pancreatic cellular data (including endocrine pancreas), we used complete pancreas tissues without the isolation of islets for single-nucleus RNA-sequencing (snRNA-seq) in order to account for the mechanism of pancreatic injury in PIGinH11 pigs. Subsequently, we compared our data with healthy humans or T2DM patients to provide support for the use of PIGinH11 pigs in metabolic disorder research.

## 2. Results

### 2.1. Serological Parameters Indicating the Metabolic Disorders in PIGinH11 Pigs

The serum biochemical parameters related to the metabolism homeostasis of glucose and lipids between the PIGinH11 and WT pigs after 12 months of dietary intervention are listed in Table 1. The results showed that the insulin, total cholesterol (TC), and the high-density lipoprotein (HDL-C) levels were significantly lower in the PIGinH11 group than in the WT group. Moreover, the increasing glucose trend was observed in the PIGinH11 group (*p* = 0.067). Glucagon was not significantly different between the two groups. Thus, compared to the WT pigs, the PIGinH11 pigs exhibited reduced insulin and increased glucose, implying the dysfunction of islets in these pigs.

### 2.2. Pancreatic Tissue Damaged with Dietary Intervention in PIGinH11 Pigs

**Figure 1:**
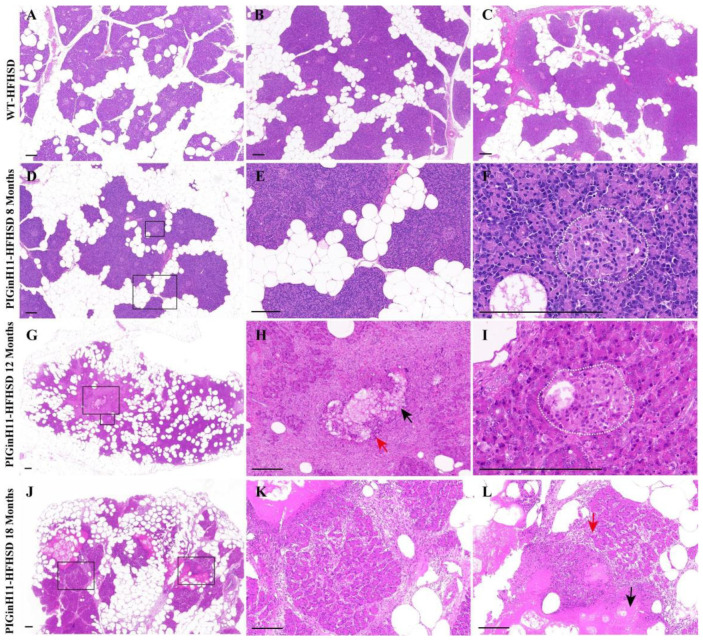
HE staining of pancreas tissues. Fat deposition in the pancreas of wild-type (WT) pigs (**A**–**C**). Fatty infiltration occurred in both inter- and intra-lobules of transgenic (PIGinH11) pigs and increased with time (**D**,**E**,**G**,**J**). The islet of the PIGinH11 pigs after 8 months of high-fat and high-sucrose diet (HFHSD) is shown in the white dashed line (**F**). The pancreas of the PIGinH11 pigs after 12 months of HFHSD shows parenchymal tissue necrosis, inflammatory infiltration (**H**). The islet of the PIGinH11 pigs after 12 months of HFHSD is shown in the white dashed line, which shows cell swelling (**I**). Pancreatic necrosis and fibrosis became more severe in PIGinH11 pigs as the HFHSD time increased (**K**,**L**). The view in the black box is enlarged for the last two figures in the same row. Red arrows point out immune cells, and black arrows show parenchymal tissue necrosis. Scale bars, 200 μm.

After 12 months of dietary intervention, there was little intralobular fat deposition in the pancreas of WT pigs (Figure 1A–C), which is consistent with the results of the 23-month HFHSD intervention [17]. In contrast, the fat infiltration can be observed in the interlobular area of the pancreas in the PIGinH11 pigs after just 8 months of dietary intervention (Figure 1D,E), and it can be seen that the fat deposition increased in both the inter and intra lobules at the following experimental stages. Up to 12 months, the ratio of the intralobular fat deposition was more than half of these areas (Figure 1G). Some parts of the parenchymal exocrine tissue appeared to have necrosis and inflammatory infiltration (Figure 1H). The cells within the islets of the PIGinH11 pigs appeared swollen (Figure 1I). It is also worth noting that after the 18-month HFHSD intervention, PIGinH11 individuals showed more severe fat necrosis (Figure 1J), fibrosis (Figure 1K), and inflammatory infiltration (Figure 1L). These results demonstrate that the PIGinH11 pigs were more susceptible to energy-overload-induced fat deposition in the pancreas, and that this irreversible damage affected the pancreatic function in PIGinH11 pigs.

**Figure 2:**
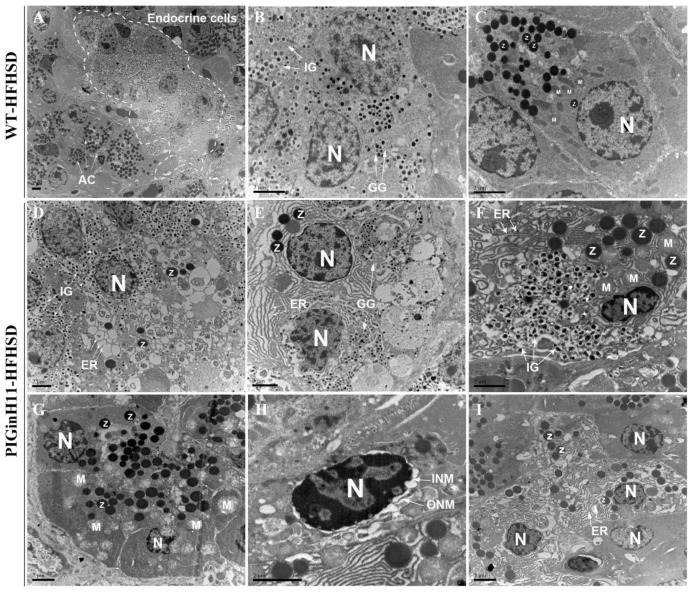
Transmission electron microscopy (TEM) photograph of wild-type and PIGinH11 pigs. An overview of the pancreas in wild-type pigs shows that they were in a healthy condition (**A**). Endocrine pancreas cells are shown in the white dashed line. The details of the normal endocrine cells (**B**) and acinar cells (**C**) are shown for wild-type pigs. Endocrine cells show endoplasmic reticulum (ER) damage (**D**–**F**). Alpha and beta cells contain dense zymogen granules close to endocrine granules in PIGinH11 pigs (**E**,**F**). Acinar cells show mitochondria swelling (**G**), outer and inner nuclear membrane separation (**H**), and ER damage (**I**). AC: acinar cell, N: nucleus, IG: insulin granules, GG: glucagon granules, Z: zymogen granules, ER: Endoplasmic reticulum, M: Mitochondria, ONM: Outer nucleus membrane, INM: inner nuclear membrane. Scale bars, 2 μm.

The transmission electron microscopy (TEM) scans of the pancreatic tissue were compared between the PIGinH11 and the WT pigs having undergone 12 months of dietary intervention. The endoplasmic reticulum (ER) was smooth and flat, and the mitochondria showed a normal shuttle shape in the WT group (Figure 2A–C). The beta cells from the PIGinH11 pigs exhibited ER dilatation and cyclization (Figure 2D). We also observed some double positive cells (individual cells containing both zymogen granules and insulin granules or glucagon granules) (Figure 2E,F). This may be related to the plasticity between the acinar and endocrine cells. Acinar cells from PIGinH11 pigs contain the swollen mitochondria (Figure 2G), separated inner and outer nuclear membranes (Figure 2H), and enlarged endoplasmic reticulum gaps (Figure 2I). This observation reveals that both the endocrine and exocrine cells of the PIGinH11 pigs were damaged at the organelle level, which in turn, affected the physiological function of the pancreas.

### 2.3. Single-Nucleus RNA-Sequencing Reveals Characterization of Pig Pancreatic Cell Types

**Figure 3:**
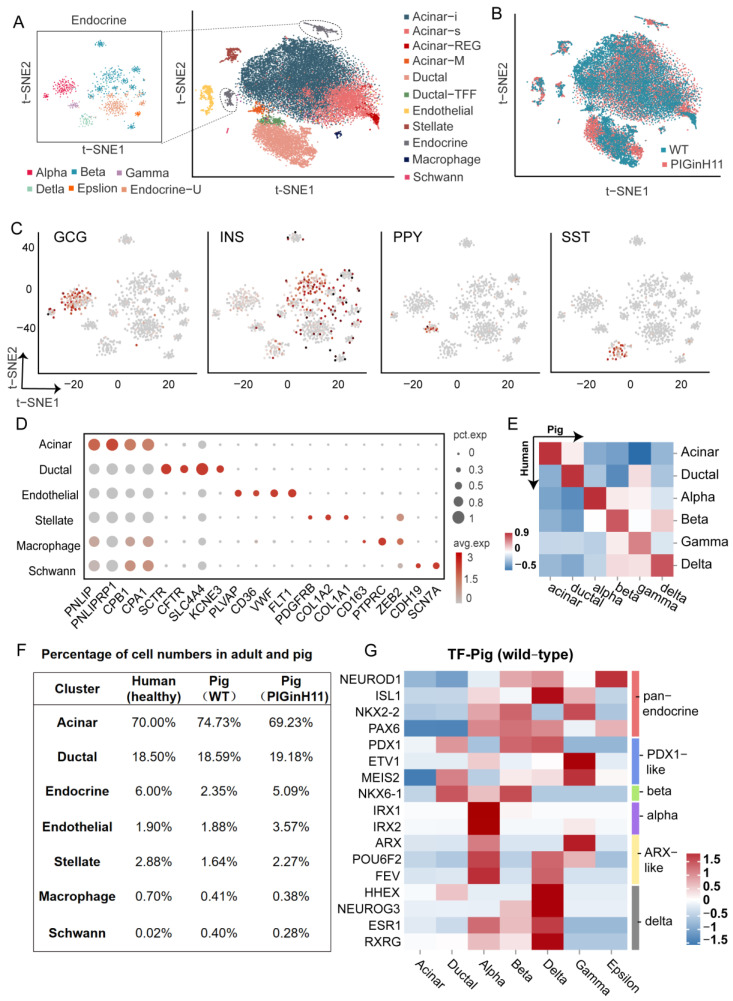
Characterization of pig pancreatic cell types. Major cell types identified from snRNA-seq of pig pancreas are shown as clusters in a two-dimensional t-SNE embedding (**A**). Percentage of cell numbers in the two groups are shown in a t-SNE map (**B**). The expressions of the marker genes of endocrine cells are shown in the t-SNE map, The color of the dot indicates the expression level of the gene in that cell, the redder the color, the higher the expression level (**C**). Other cluster marker genes are shown in a bubble map (**D**). Comparison of correlations between species of each cell cluster (**E**). Percentage of cell numbers in healthy adults and pigs (**F**). Heat map of expression patterns of endocrine pancreatic function in WT pigs (**G**).

**Figure 4:**
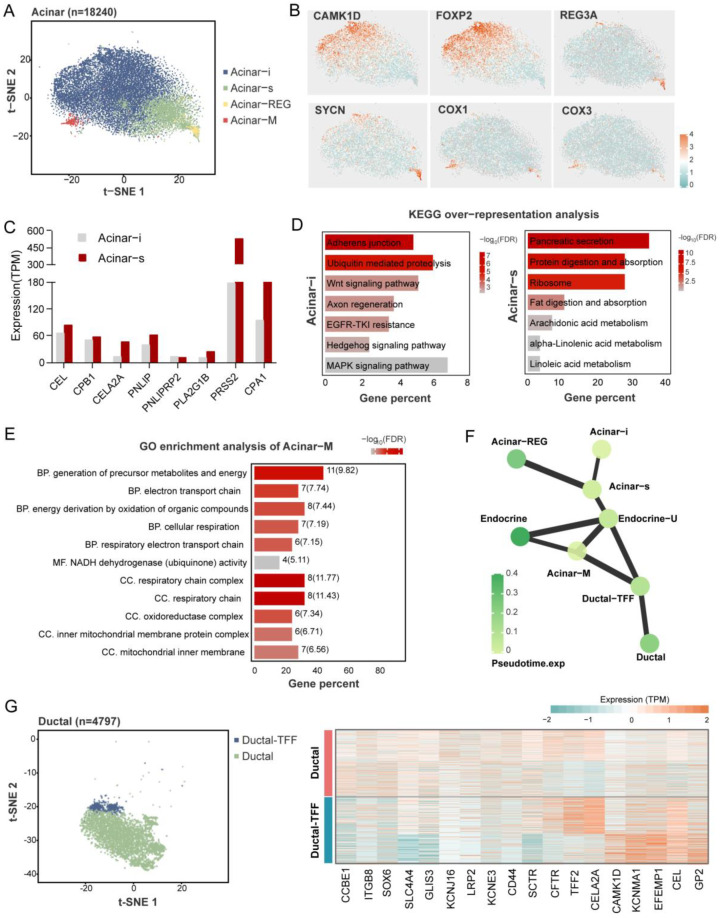
Heterogeneity of pancreatic exocrine cells. An overview of acinar cells cluster in a t-SNE map (**A**). Marker genes of each acinar subcluster are shown in a t-SNE map (**B**). The expression (TPM) level of digestive enzymes in acinar-i and acinar-s clusters (**C**). Kyoto Encyclopedia of Genes and Genomes’ (KEGG) over-representation analysis of upregulated genes in Acinar-i and Acinar-s clusters (**D**). Gene ontology (GO) enrichment analysis of Acinar-M cluster. BP means biological process, MF means molecular function, and CC means cellular component. The numbers at the back of the bars show the gene numbers enriched in the pathways (**E**). PAGA abstracted graph represents the similar relationship between cell clusters. The shade of the dot color indicates the state of its pseudo-time time; the darker the color the later the time. A thicker line between the dots shows a stronger similarity between the two cell clusters (**F**). Characteristics of the ductal cell subclusters. An overview of ductal cells cluster in a t-SNE map (**left**), and the heatmap shows key genes expression level in two ductal clusters (**right**) (**G**).

To further investigate the pathological changes in the pancreas of PIGinH11 pigs at the cellular and molecular levels, we conducted single-nucleus transcriptome sequencing on the pancreas of PIGinH11 (*n* = 1) and WT (*n* = 1) pigs having undergone 12 months of dietary intervention. After data quality control and gene expression quantification, a total of 25,383 nuclei of a high quality were subjected to the proceeding analysis, including 12,133 nuclei in the WT group and 13,250 nuclei in the PIGinH11 group. More details of the quality control process are shown in Table S1 and Figure S1. All the cell gene matrices from the two samples were imported into the Seurat for cell clustering. In total, 16 separate cell clusters were identified using a semi-supervised cell annotation method (Figure 3A). These clusters included the exocrine pancreas (acinar and ductal cells), endocrine pancreas (alpha, beta, delta, gamma, epsilon cells, and an uncharacterized endocrine cluster named Endocrine-U), pancreatic stellate cells, macrophages, endothelial cells, and Schwann cells. Both the PIGinH11 and WT groups contributed equally to most cell clusters, except for the endothelial and endocrine cells (Figure 3B,F). Firstly, the endocrine cells consisted of five types of cells, which are characterized by the expression of specific marker genes, including GCG (alpha cells), INS (beta cells), PPY (gamma cells), and SST (delta cells), as shown in Figure 3C. Strikingly, we identified rare epsilon cells that highly expressed GHRL (Figure S2A). Notably, we found a unique cluster (Endocrine-U), which did not strictly express any marked hormone that was like other endocrine cells (Figure S2A). Moreover, the correlation analysis between each cluster indicated that those cells were highly similar to beta cells, epsilon cells, and acinar-i cells (Figure S2B). Subsequently, we performed PAGA analysis to rank the low-dimensional projection positions of the cells based on the similarity and dynamic change characteristics of the gene expression patterns, which shows the similarity between clusters. The Endocrine-U cluster was intermediate between the exocrine and endocrine cells (Figure 4F). This cluster might be a class of transition-state cells.

As for the exocrine pancreas, most identified cells were acinar cells, characterized based on the specific expression of digestive enzymes, such as PNLIP and CPB1. These data showed heterogeneity within acinar cells, including four special acinar clusters, idling acinar cell (Acinar-i), secretory acinar cell (Acinar-s), Acinar-REG (high expression of regenerating family member genes) and Acinar-M (high expression of mitochondria-related genes). Ductal cells highly expressed the classical markers CFTR, SLC4A4, and KCNE3. Two distinct ductal clusters, Ductal and Ductal-TFF (high expression of genes related to mucus secretion), were identified, consistent with prior studies [9]. The pancreatic stellate cells were highly expressed PDGFRB, COL1A1, COL1A2, and macrophage, which were characterized by the expression of CD163, PTPRC, and ZEB2, and endothelial cells expressing FLT1, PLVAP, and VWF. We also detected a cluster of Schwann cells expressing CDH19 and SCN7A (Figure 3D). The marker genes are shown in Table S2. All these marker genes were used in human ScRNA-seq studies. The correlation analysis of the counterpart cell clusters between the species with a z-score supported these reliable cell annotations and confirmed the high level of similarity of the key gene expression patterns between humans and pigs (Figure 3E).

### 2.4. Cell-Type-Specific Expressing Genes in Each Cell Cluster

Almost each cluster cell number percentage in our data was similar to humans [11]; the cell cluster with the largest percentage in the porcine pancreas was the acinar cells (about 70%), followed by the ductal cells (about 18%). The percentage of endothelial cells was also similar in humans and pigs (about 1.9%). However, the cell percentage of the pancreatic stellate cells was approximately twice as high in healthy humans (2.88%) as in WT pigs (1.64%). The proportion of endocrine cells in healthy humans was about 6% in that report, which may be related to their pancreatic sampling site and age, with a large inter-individual variation. In WT pigs, the proportion was only 2.35% (Figure 3F). Subsequently, we focused on the expression of the transcription factors related to pancreatic function in various populations of endocrine cells and compared them with human data. There were several reported transcription factors (TFs) related to pancreatic biology in healthy humans that have similar expression patterns in pigs, such as NEUROD1, PAX6 (pan-endocrine cells), PDX1 (beta and delta cells), IRX1, IRX2 (alpha cells), ETV1 (gamma cells), ARX (alpha and gamma cells), and HHEX (delta and ductal cells) [8,18]. Moreover, some unique TF expression patterns in pigs were identified from our data, including NKX6-1 (ductal, alpha, beta cells), ESR1, and RXRG (alpha, beta, and delta cells), which are beta-cell-specific transcription factors in healthy humans. Some transcription factors with PDX1-like functions, ETV1 and MEIS2, exhibited strong gamma-cell specificity. POU6F2 was detected in alpha, gamma and delta cells, and FEV was found to act as an alpha- and delta-specific TF (Figure 3G). This shift in the expression specificity suggests that although the overall pancreatic function was somewhat conserved across the species, specific expression across cell types was possibly plastic and inconsistent across species.

After cell annotation using classical marker genes for each cell cluster (Table S2), several additional genes were expressed especially high (|logFC| > 3, FDR < 0.01) in each cell cluster in the porcine pancreas (File S1). RBPLJ had high levels of expression in the acinar cells, which play a regulatory role in the development and function of acinar cells. PKHD1 was expressed in most ductal cells (99% of all ductal cells), and its expression was second only to SLC4A4, a classical marker of ductal cells (File S1). PTPRB, FGD5, and EGFL7 previously served as marker genes for endothelial cells in non-pancreatic tissues, and they were exclusively expressed in porcine pancreatic endothelial cells. In pancreatic stellate cells, PRKG1 was used as a marker gene for mesenchymal cells in the pancreas or liver; pancreatic stellate cells are a type of mesenchymal cell (Figure S3). These genes may have been the marker genes for the target cluster in the porcine pancreas.

### 2.5. Heterogeneity of Pancreatic Exocrine Cells

Like the prior study, we found that the heterogeneous subtypes of acinar and ductal cells were based on marker gene expression patterns. Four clusters of acinar cells were identified in the pig (Figure 4A), including the secretory acinar cell (Acinar-s), the idling acinar cell (Acinar-i), the high expression of the regenerating family member gene acinar cell (Acinar-REG), and the increased expression of the mitochondria-related gene acinar cell (Acinar-M). Among them, two acinar clusters with typical exocrine functions, Acinar-s and Acinar-i, had the highest proportions. The Acinar-i comprised the most significant part of the acinar cells; these cells experience the increased expression of CAMK1D and FOXP2 (Figure 4B), and are enriched with KEGG pathways that are related to cell proliferation and differentiation, such as the “Wnt signaling pathway”, “MAPK signaling pathway”, and “Hedgehog signaling pathway” (Figure 4C). The Acinar-s was characterized by the high expression level of digestive enzymes such as PRSS2, CPA1, and PNLIP (Figure 4D). Differential gene enrichment analysis among the acinar clusters showed the enrichment of the “Pancreatic secretion”, “Protein digestion and absorption”, and “Ribosome” pathways, which demonstrate that acinar-s cells synthesize and secrete digestive enzymes at a high rate (Figure 4C). As for the expression of digestive enzymes, that of the the Acinar-s cluster was two times higher than that of Acinar-i, implying that Acinar-i may be less secretory but more involved in cell signaling pathways and might be stimulated to differentiate into mature acinar cells. Acinar-REG expressed higher levels of the mRNA-encoding regenerating islet-derived family member 3 protein and syncollin (Figure 4B). In previous snRNA-seq studies, Acinar-REG cells were represented by linking them to pancreatic cancer and pancreatic ductal adenocarcinoma (PDAC). Notably, we found a unique cluster named Acinar-M, which is characterized by the high expression of genes associated with mitochondria, such as COX1 and COX2. The GO enrichment pathway is also relevant to the electron respiratory chain and oxidative phosphorylation (Figure 4E). Based on PAGA analysis, the Acinar-M cells may be adenoidal cells at the node of acinar and endocrine cell differentiation (Figure 4F and Figure S2).

There were two heterogeneous clusters of ductal cells, Ductal and Ductal-TFF. The Ductal population (80% of ductal cells) exhibits higher expression levels of classical ductal markers such as CFTR, SLC4A4, and SCTR. The Ductal-TFF cluster, which has a relatively small cell number, was characterized by the high expression of genes related to mucus secretion, such as TFF2 (Figure 4G). It is likely that it protects ductal cells from damage caused by digestive enzymes. The availability of these two types of ductal cells in humans and mice has been demonstrated, suggesting that they are conserved between species.

### 2.6. Impaired Beta Cell Function in PIGinH11 Pigs

**Figure 5:**
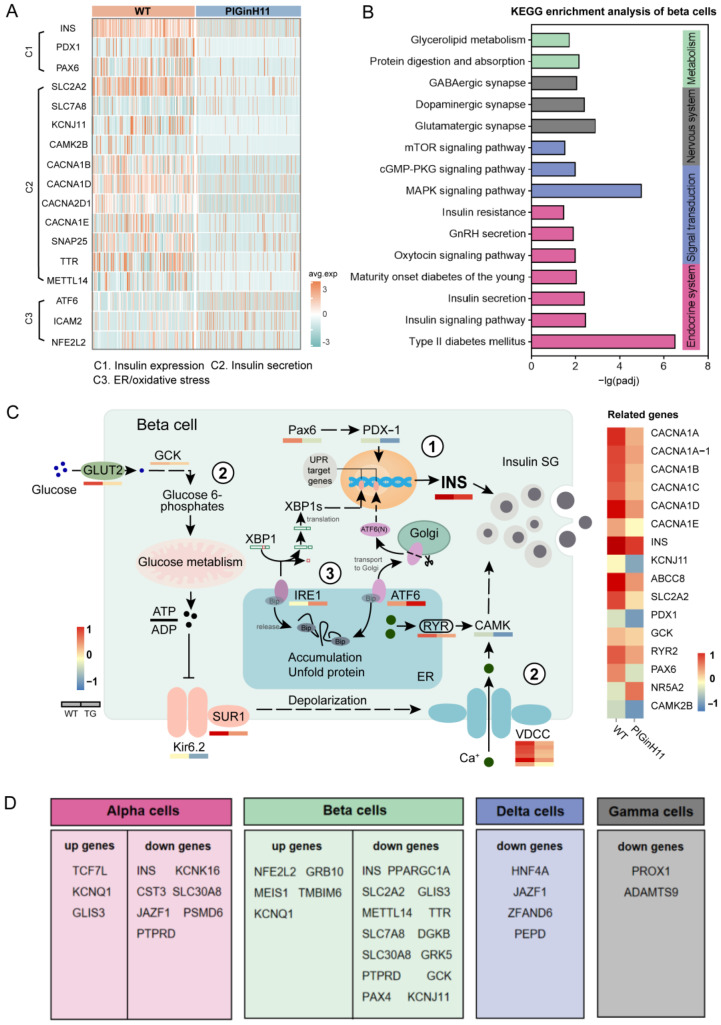
Alteration of T2DM-related genes and beta cell dysfunction in PIGinH11 pigs. Key genes that alter significantly in the PIGinH11 group (**A**). The three processes were as follows: C1: insulin expression, C2: insulin secretion, and C3: ER/oxidative stress. KEGG’s over-representation analysis of DEGs in beta cells (**B**). Pattern diagram of the beta cell dysfunction pathway, including the following three pathways (**C**): 1. Decreased insulin expression. The mRNA levels of INS and regulating insulin produce the genes deduced. 2. Decreased insulin secretion. The genes related to the glucose-stimulated insulin secretion test (GSIS) process, such as SLC2A2, GCK, KCNJ11, and ABCC8, show a decreased expression. 3. ER stress. Gene expressions linked with unfolded protein response (UPR) are increased. The expression levels of the mentioned genes are shown in the below and right heatmaps. The T2DM-related genes that undergo the same changes in T2DM patients and PIGinH11 pigs are shown in table (**D**).

Since serum insulin levels were significantly lower in PIGinH11 pigs, demonstrating reduced beta cell function, we focused our analysis on the gene expression pattern of beta cells. Notably, the expression of INS was significantly decreased in PIGinH11 pigs (Figure 5A); it was seven times higher in WT pigs than in PIGinH11 pigs. Moreover, PIGinH11 pigs exhibited a decrease in the number of cells as a percentage of beta cells (Table S3). In addition to the differential expression of INS, we identified 513 differentially upregulated and 1008 downregulated genes in the beta cells (Table S4), which were mainly involved in insulin production, secretion and cell damage (Figure 5A). Subsequently, the four main pathway types for the KEGG enrichment analysis of intergroup DEGs in beta cells are shown in Figure 5B. The first type was related to the endocrine system, including the pathways of “Type II diabetes mellitus” and “Insulin signaling pathway”. The second type was associated with signal transduction, such as the “MAPK signaling pathway” and the “cGMP-PKG signaling pathway”. The remaining two types were nervous system and metabolism-related, including “Glutamatergic synapse” and “Protein digestion and absorption”. This result indicates an abnormal state of insulin synthesis and secretion and intercellular communication in the beta cells of the PIGinH11 pigs.

An in-depth study of the beta cell intergroup differential analysis revealed that beta cell dysfunction might occur at the transcriptome level in PIGinH11 pigs via three pathways. The first one sees a reduction in insulin expression, which is manifested by the reduced expression level of insulin genes (INS), as well as the significantly reduced expression of genes such as PAX6 and PDX1, which are upstream regulators of insulin expression. The second sees a decrease in insulin secretion, as evidenced by a significant downregulation of the mRNA levels of key genes involved in classical insulin secretion in beta cells, such as the transporter protein that mediates glucose entry into cells (SLC2A2), the rate-limiting enzyme in glucose metabolism (GCK), the potassium channel protein (KCNJ11, ABCC8) and calcium voltage gating (CACNA1A, B, D, E). The third sees the appearance of endoplasmic reticulum stress, as evidenced by the significant upregulation of the expression of marker genes of endoplasmic reticulum stress, such as ATF6, and ICAM2 (Figure 5C).

Then, compared with those currently reported to be associated with T2DM [6,9,10], the DEGs obtained in each endocrine cell were found to be consistent with humans (Figure 5D). Five identical upregulated DEGs and 14 identical downregulated DEGs were found in beta cells and had the same expression pattern in both pigs and T2DM patients. According to their reported function, these involved genes were divided into three main categories, according to their reported function. One was related to beta cell development and mass, and included MEIS1, GRB10, GLIS3. The second was associated with beta cell function, specifically regarding insulin synthesis and secretion, and included INS, KCNJ11, GCK, TTR, SLC7A8, SLC2A2, METTL14, KCNQ1, SLC30A8, and JAZF1. The last was associated with cellular stress, and includedNFE2L2 and TMBIM6. Moreover, several DEGs in other endocrine cells were found to be mainly involved in cellular damage and pancreatogenesis, such as JAZF1, HNF4A, and PROX1. Other endocrine cells also play an important role in regulating glucose homeostasis, and their abnormal state also affects beta cell function, so the detected common DEGs may be important targets in regulating the course of T2DM.

### 2.7. Acinar Cell Dysfunction in PIGinH11 Pigs

**Figure 6:**
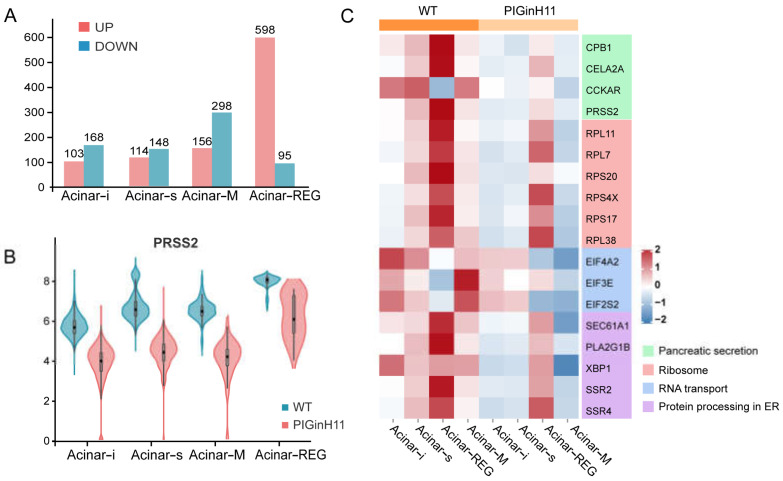

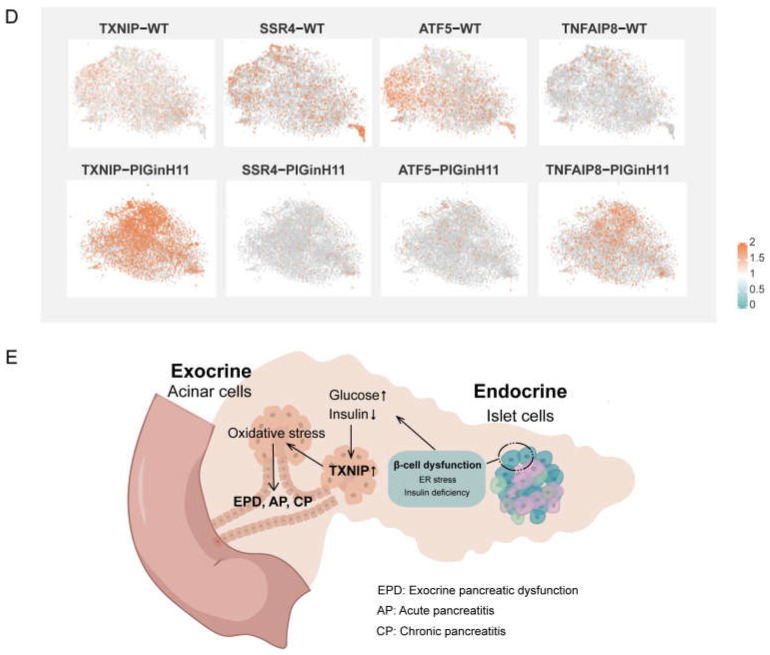
Functional alteration of acinar cells in PIGinH11 pigs. Histogram of the number of differential genes between subclusters of each acinar cell (**A**). Red indicates differential genes upregulated in PIGinH11 group. Blue indicates differential genes downregulated in PIGinH11 group. Violin plot of trypsinogen 2 (PRSS2) depicts the expression between the two groups of each acinar cell (**B**). Red indicates PIGinH11 pigs, and blue indicates WT pigs. Heat map of genes differing among distinct groups in acinar cells (**C**). Genes associated with ribosomes are in red, RNA transport is in blue, protein synthesis in the endoplasmic reticulum is in purple, and pancreatic secretion is in green. Expressions of key genes in acinar cells in both groups are shown in t-SNE map (**D**). The hypothesis model of how endocrine pancreas damage affects exocrine pancreas function (**E**). Beta cell dysfunction causes insulin to decrease and glucose to increase. Furthermore, it increases the mRNA levels of TXNIP in acinar cells. The high expression of TXNIP caused oxidative stress and resulted in cell damage. The cellular stress state is a prerequisite for many pancreatic diseases (EPD, AP, CP).

The exocrine pancreas assumes the role of producing and delivering digestive enzymes. As the group of cells with the highest protein synthesis, it is susceptible to functional changes in response to external stimuli or changes in molecular signals, even leading to pancreatitis or pancreatic cancer. The results of the intergroup difference analysis showed that the function of the PIGinH11 acinar cells changed; the four subpopulations of Acinar-i, Acinar-s, Acinar-REG, and Acinar-M identified 103, 114, 156, and 598 upregulated DEGs and 168, 148, 298, and 95 downregulated DEGs, respectively (Figure 6A). The expression level of digestive enzymes significantly decreased in four acinar cell clusters, especially in the trypsinogen gene PRSS2, which dominated the digestive enzyme component of pancreatic juice (Figure 6B). Then, we found that genes involved in protein synthesis processing and secretion were significantly reduced in all four groups of acinar cells; these included ribosomal genes, such as RPL11, RPL7, RPS20, transcription initiation factors, such as EIF4A2 and EIF3E, and protein transport protein genes, including SEC61A1 and SEC62 (Figure 6C). This result indicates that acinar cells may have reduced the expression and secretion of digestive enzymes, especially proteases. In addition, several genes related to the cellular stress response were of particular interest, such as TXNIP, SSR4, ATF5, TNFAIP8 (Figure 6D), these genes play an essential role in ER and oxidative stress. Overall, acinar cells from PIGinH11 pigs showed dysfunction, particularly in regard to digestive enzyme insufficiency and organelle stress.

## 3. Discussion

In a previous study, we performed a short-term diet-based (12 weeks) intervention on PIGinH11 pigs [16], which showed impaired glucose tolerance and varying degrees of pancreatic, hepatic, and adipose damage in PIGinH11 pigs. To investigate the pathological characteristics of the pancreas in PIGinH11 pigs and to assess its feasibility as an animal model of metabolic disorders, we evaluated the pathological histology of PIGinH11 and wild-type pigs when subjected to different durations of HFHSD and a selected 12-month dietary intervention in order to conduct snRNA-seq analysis. With the dietary intervention, PIGinH11 pigs suffered insulin deficiency and severe fatty infiltration in the pancreas. Meanwhile, as the induction time increased, PIGinH11 pigs showed fibrosis, inflammatory infiltration, and fat necrosis in the pancreas. We found that two human disease susceptibility genes (GIPR^dn^ and hIAPP) could cause impaired insulin secretion, beta cell ER stress, and apoptosis [19]. This situation is a pathological condition similar to PIGinH11, indicating that PIGinH11 pigs are a suitable large animal model that can be used to study metabolic disorder-related diseases.

Herein, a comprehensive single-cell level atlas of the pig pancreas (including endocrine glands) was constructed. We identified 16 clusters of pancreatic cells by using the classical marker genes utilized in the previous article [6,7,8,9,10,11,12]. At the same time, heterogeneous subclusters of acinar cells and ductal cells were also present in our data and were similar to those found in humans, such as acinar-REG, acinar-s, acinar-i, and ductal-TFF [8,11]. We also identified a unique group of acinar cells (Acinar-M). This cluster was characteristically highly expressed in mitochondria-related genes, while their high similarity to ductal cells and beta cells placed them in the intermediate of the three major cell types. A cluster of endocrine cells that shares commonality with Acinar-M is the Endocrine-U cluster, which was strongly associated with beta cells. On the other hand, genes regulating the development of acinar cells, such as NR5A2 and RBPJL, were characteristically upregulated within this cluster (Figure S2C,D) [20]. Assuming that Acinar-M is a group of middle-state-oriented acinar cells, the Endocrine-U cluster is a group of intermediate-oriented endocrine cells. These two types of cells may correspond to the double positive cells when exposed to insulinogen, glucagon zymogen and digestive zymogen particles that we captured under TEM (Figure 2E,F). A previous study has also mentioned the presence of such double positive cells [21]. However, the exact role of such cells in the pancreas still needs further study. Most pancreatic cell populations in our data were similar to humans, such as acinar cells, ductal cells, and endothelial cells [11]. Moreover, comparing these transcriptional-based data to healthy humans, more similar features were observed. There were several reported transcription factors (TFs) related to pancreatic biology in healthy humans that were found to have similar expression patterns in pigs, such as NEUROD1, PAX6, PDX1, etc. [8,18]. Therefore, we assume that pancreatic cell characteristics are conserved to a large extent between pigs and humans.

Pancreatic beta cell dysfunction is a primary pathogenic cause of T2DM. In turn, beta cell dysfunction is associated with a lack of insulin secretion and the loss of beta cell mass [22]. At the single-cell transcriptome level, specific molecular processes that impaired the function of the PIGinH11 group beta cells were identified. These include a decrease in the proportion of beta cells, an increase in the proportion of intermediate state cells (Endocrine-U cluster), a reduction in the level of transcription factors characteristic of mature beta, a lack of insulin mRNA, and a decrease in the expression of key genes in the glucose-stimulated insulin secretion (GSIS) process. The studies conducted inT2DM patients and T2DM mouse models showed that the occurrence of some mature beta cell function-related TFs in beta cells is deficient, such as in PDX1 [23]. The deficiency of these TFs contributes to the loss of functional beta cell numbers. Beta cells are susceptible to ER stress in metabolic disturbance [24]. Moreover, ER stress could induce an unfolded protein response (UPR) [25], and a chronic UPR state can contribute to beta cell death and diabetes [26]. Corresponding to the beta cell endoplasmic reticulum damage we observed under TEM (Figure 2F), some genes related to the UPR were elevated at the mRNA level. These indicate that the PIGinH11 group was in a state of diet-induced beta cell dysfunction.

Meanwhile, we also refer to several articles investigating the alterations made to gene expression characteristics in T2DM [6,9,10], and then identified several T2DM-related genes with an altered expression in endocrine cells, especially in beta cells. MEIS1 expression was significantly elevated in T2DM patients and the PIGinH11 group, and transcriptionally regulates both PDX1 and PAX6 [27]. Other genes with the same expression pattern are NFE2L2 and GRB10. NFE2L2 is an essential regulatory element for the onset of oxidative stress in vivo and has also been demonstrated to prevent pancreatic beta cell injury [28]. GRB10 was identified as a pivotal regulator of beta cell dedifferentiation and mass [29]. The thyrotropin transporter protein (TTR) is a functional protein in pancreatic beta cells and promotes insulin release and prevents beta cell death [30]. The tetramer of the TTR has a positive role in the pancreatic beta cell stimulation–secretion coupling. It enables the glucose-induced elevation of cytoplasmic-free Ca^2+^ concentration and increases insulin release [31]. TTR KO mice impaired the recovery of blood glucose and glucagon levels [32]. A beta cell-specific METTL14-KO mouse exhibits poor glucose tolerance, reduced glucose-stimulated insulin secretion, increased beta cell death and reduced beta cell mass [33]. These genes might be valuable targets in T2DM disease research and treatment.

In clinical settings, EPD that is characterized by the inadequate secretion of digestive enzymes has been observed frequently in diabetic patients [6]. Despite this high prevalence, the pathophysiology of EPD in diabetes is not fully understood; until now, only the diminished nutritional effects of insulin, fibrosis and steatosis, and inflammation have been established [34]. A comparable phenotype during diet induction was presented in PIGinH11 pigs (Figure 1). Notably, inactive levels of protein synthesis and deficient digestive enzyme production were exhibited in the acinar cells. Moreover, the endoplasmic reticulum and mitochondria appeared to be damaged in acinar cells (Figure 2G,I). In addition, the TXNIP was significantly increased in the acinar cells of the PIGinH11 group. Notably, we also found that the TXNIP expression was increased in the RNA-seq data of PIGinH11 pigs after 12 months of diet induction when compared with 2 months (unpublished data). TXNIP is a major regulator of glucose homeostasis and a responder to oxidative stress. Circulating levels of TXNIP are dramatically elevated in T2DM patients [35]. Numerous studies have demonstrated that TXNIP is induced by high glucose and promotes beta cell apoptosis [35,36]. We hypothesize that the elevated glucose and insulin deficiency due to beta cell dysfunction may promote the increased expression of TXNIP in acinar cells, which causes cellular damage via oxidative stress (Figure 6E). However, it has not been reported how TXNIP acts in acinar cells. The relationship between TXNIP and cellular stress in pancreatic exocrine cells needs to be further investigated.

There are still some limitations to this study. Firstly, the Seurat used for our analysis was based on the V3 version, which may have generated varied results as the version was updated. Secondly, a larger sample number for sequencing might have yielded a more comprehensive global pattern. Thirdly, this study did not involve a validation experiment, and more research was needed to illustrate the potential damage of molecular mechanisms to the pancreas.

## 4. Materials and Methods

### 4.1. Animal

A total of 6 transgenic males (PIGinH11 group) and 6 age-matched wild-type males (WT group) were used. Pigs were fed with a control diet until 6 months and with HFHSD (37% sucrose, 53% control diet and 10% pork lard) for the next 8 months (one individual sampled for histopathological examination for both groups), 12 months (four individuals) and 18 months (one pig for prolonged intervention). Each experimental animal lived in a single cage, was fed twice a daily, and was given access to water. The ambient temperature was kept at 16–28°, with humidity at 40–70%. We used two groups of pigs in the research. All animal experiments were approved by the Animal Care and Use Committee of the Institute of Animal Sciences, Chinese Academy of Agricultural Sciences (No. IAS2019-12). Each experimental procedure was performed under the Guide for the Care and Use of Laboratory Animal, IAS, CAAS. At the end of the HFHSD intervention, the animals were sacrificed humanely. A part of the tissue, about 1 mm, was promptly mounted in an electron microscope fixative (glutaraldehyde, 4%). One part of the tissue was frozen immediately in liquid nitrogen, and the other was filled with 4% PFA for fixation.

### 4.2. Serum Test

After overnight fasting, a total of 20 mL of venous blood was obtained from the anterior vena cava of each pig and placed in a BD vacutainer tube (KJ030AS, Kangjian, Taizhou, China). Whole blood was centrifuged at 4 °C 3000 rpm for 10 min to separate the serum after 1 h at ambient temperature. The serum concentration of the total cholesterol (TC) and high-density lipoprotein cholesterol (HDL) was measured using an AU480 auto-analyzer (Olympus Co., Tokyo, Japan). The fasting blood glucose level was measured using blood glucose meters (Johnson, Ultra, New Brunswick, NJ, USA) and the venous blood of each pig’s ear. The serum levels of insulin (10-1200-01, Mercodia, Uppsala, Sweden), glucagon (10-1281-01, Mercodia, Uppsala, Sweden), and C-peptide (DY2648, R&D, Minneapolis, MN, USA) were measured using an enzyme-linked immunosorbent assay and the corresponding antibodies.

### 4.3. Histopathological Examination

After the pigs were sacrificed, the pancreas tissues were immediately fixed in 4% paraformaldehyde overnight. Then, these fixed tissues were dehydrated, embedded in paraffin, and sliced into continuous sections. The sections were stained with hematoxylin and eosin (HE) in order to assess the pancreas’s pathological state. Histological sections were scanned and read in panoramic view using CaseViewer software (v2.4.0). For the transmission electron microscopy samples, a large volume of approximately 1 cubic millimeter of tissue block was removed from each pancreas and quickly placed in glutaraldehyde, 4% (EM Grade), for fixation. The samples were stored at 4 °C for 12 h and then placed in osmium tetroxide for secondary fixation. Then, they were gradient dehydrated and embedded in fresh epoxy 618. Ultrathin sections (60–80 nm) were cut and stained with lead citrate, followed by a Phillips CM-120 transmission electron microscope examination.

### 4.4. Single-Nucleus RNA-Sequencing

One pancreatic tissue piece was taken from each group of pigs from a similar location (body of the pancreas). Sample preparation, nucleus isolation, library preparation, and sequencing were performed using Gene Denovo Biotechnology Co., Ltd. (Guangzhou, China) following the guidelines of 10× Genomics (10× Genomics, Pleasanton, CA, USA). Briefly, nucleus suspensions were loaded on a 10 X Genomics GemCode single-cell instrument, and libraries were generated using the cDNAs with Chromium Next GEM Single Cell 3′ Reagent Kits v3.1. The libraries were ultimately sequenced with an Illumina HiSeq 4000 instrument. Cell Ranger (v3.1.0) was used to perform data quality statistics on the raw data. Reads with low-quality barcodes and UMIs were filtered out, and then were aligned to the reference genome (Sus Scrofa 11.1). Before quantification, the UMI sequences were corrected for sequencing errors, and valid barcodes were identified based on the EmptyDrops method. The cell by gene matrices were produced via UMI counting and cell barcodes calling, then the gene matrices for each sample were individually imported to Seurat (v3.1.1) for downstream analysis. Before downstream analysis, we removed doublets by DoubletFinder (v2.0.3), and cells with gene counts of 270-3700 per cell, UMIs with counts lower than 1300 per cell, and cells with a percentage of mitochondrial genes per cell that was less than 10% were screened for downstream analysis. After retaining high-quality cells, expression was normalized using a common “log homogenization” method. Following data merging using Harmony, principal component analysis (PCA) was conducted and the best principal component was selected for subsequent analysis. Based on the previously identified principal components, the Euclidean distance between cells was calculated. Then, Seurat embedded cells in a SNN (shared-nearest neighbor) graph based on the Euclidean distance between the cells. Next, the SNN graph was partitioned into highly interconnected quasi-populations according to the Jaccard distance between two cells in local proximity. Finally, the cells were clustered into groups using Louvain’s algorithm and the data were visualized using nonlinear dimensionality reduction (t-SNE). Subsequently, cell annotation using semi-supervised methods (singleR and manual) was performed.

We used Scanpy’s rank sum test to filter the genes with upregulated expression in each cell subpopulation. The screening criteria were the gene upregulation fold change (FC) |logFC| > 1 and *p* < 0.05, alongside gene expression in more than 25% of the cells in the target cluster. The screening criteria for differentially expressed genes between the two groups for each cell type were |logFC| > 1 and *p* < 0.05. We used OmicShare, an online tool based on the R package, to map all the peak genes to gene ontology (GO) terms or to the Kyoto Encyclopedia of Genes and Genomes’ (KEGG) pathways in the GO database “http://www.geneontology.org/ (accessed on 8 May 2022)” or KEGG database “https://www.kegg.jp/ (accessed on 8 May 2022)”. The calculated *p* values were FDR corrected to a threshold of FDR ≤ 0.05. The temporal arrangement of cells when using PAGA analysis (Scanpy v1.9.1) takes advantage of the similarity and dynamic changes in the gene expression patterns in order to arrange the low-dimensional projection positions of cells and thus reflect the differentiation characteristics between cells.

### 4.5. Statistical Analysis

The statistical analysis and mapping were performed using Prism8 and Rstudio based on R version 4.0.4. A comparison between the two groups was performed using the two-tailed Student’s *t*-test. Data were expressed as means ± SEM, and a *p*-value < 0.05 was considered significant.

## 5. Conclusions

In summary, after long-term dietary induction, PIGinH11 pigs were more sensitive to energy overload and exhibited the pathological features of insulin deficiency, fatty deposition, inflammatory infiltration, fibrosis tissue necrosis, double positive cells, endoplasmic reticulum (ER) and mitochondria damage, similar to those found in T2DM patients. Single-nucleus transcriptome analysis discovered two specific groups of cells (Acinar-M and Endocrine-U), which may be associated with the dedifferentiation or transdifferentiation of endocrine cells. An analysis of DEGs between the PIGinH11 and WT groups of each cell cluster indicated the decreased gene expression levels of insulin synthesis and secretion-related genes in the beta cells, together with an increased endoplasmic reticulum stress, suggesting an impaired beta cell function. Like clinical diabetes, which is often accompanied by EPD, organelle damage was exhibited in the PIGinH11 group of acinar cells. Large digestive enzymes were downregulated at the transcriptome level, and the upregulation of TXNIP by high glucose levels potentially promoted the dysfunction of the endocrine to exocrine cells in PIGinH11 pigs. In this paper, we constructed the first complete pig pancreatic atlas, including the endocrine, exocrine and other cells, while cross-talking with human findings, thus supporting the use of PIGinH11 pigs as an animal model of T2DM. This paper has also provided some reference targets for the research and therapy of human pancreatic dysfunction.

## Figures and Tables

**Table 1:** Serum biochemical parameters.

Group	TC (mmol, L)	HDL-C (mmol, L)	Glucose (mmol, L)	Insulin (uIU, mL)	Glucagon (pg, mL)
**WT (*n* = 4)**	3.7 ± 0.30	2.3 ± 0.19	5.35 ± 0.49	62.8 ± 5.47	114.45 ± 19.09
**PIGinH11 (*n* = 4)**	2.4 ± 0.37	1.45 ± 0.24	6.56 ± 0.23	45.57 ± 4.27	148.29 ± 18.04
***p* value**	0.033	0.034	0.067	0.048	0.245

Data are mean ± SEM, statistical difference was determine using the Student’s *t*-test.

## Data Availability

The raw sequence data have been deposited in GEO under accession code GSE226141. The code used to analyze this dataset was upload to the Github repository https://github.com/apanhui/scRNA_analysis (accessed on 4 April 2023).

## Supplementary Materials

The following supporting information can be downloaded at: https://www.mdpi.com/article/10.3390/ijms24097701/s1.
