# Supplementary material for: SnRNA-Seq of Pancreas Revealed the Dysfunction of Endocrine and Exocrine Cells in Transgenic Pigs with Prediabetes
Source: Int J Mol Sci. 2023 Apr 22;24(9):7701. doi: 10.3390/ijms24097701 (PMC10178631; doi:10.3390/ijms24097701)
Supplement: Supplementary file 1 [file ijms-24-07701-s001.zip › Supplementary Figures and Tables.pdf]

Supplementary Material

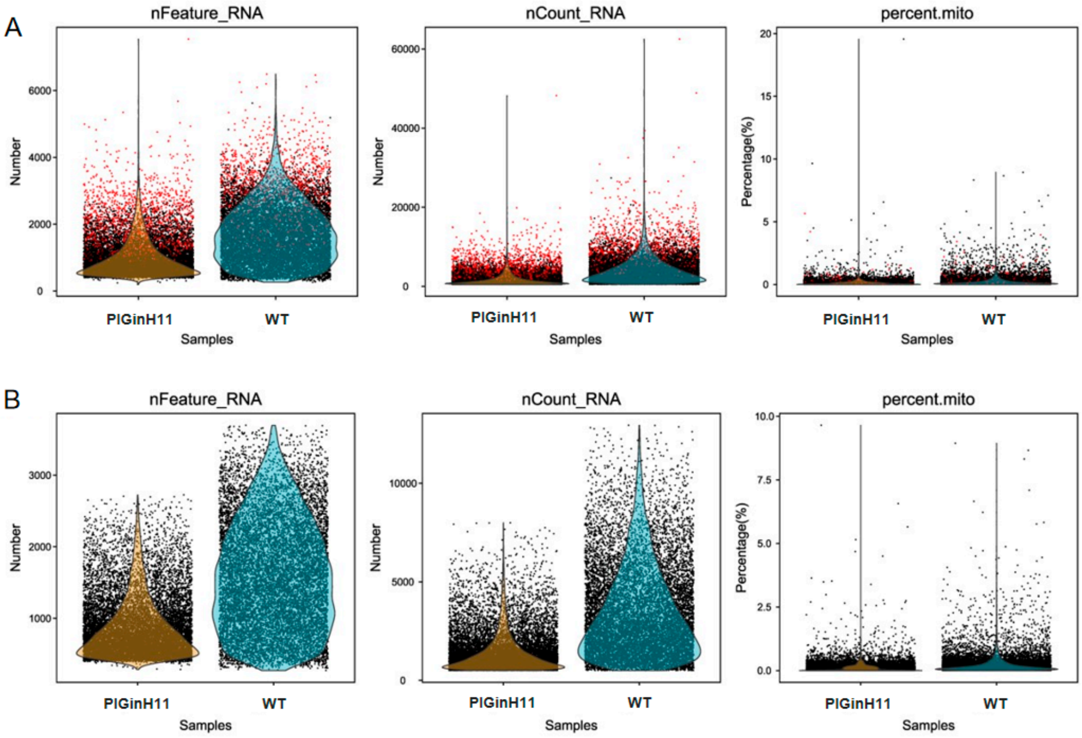

Supplementary Figure S1. Data quality information (nUMI, nGene, percentage of mitochondria genes). Basic information of each sample cell before (**A**) and after (**B**) quality control. Scattered dots in the figure represent a cell, red for multiple cells and black for single cells. nFeature_RNA represents the number of genes per cell, nCount_RNA represents the number of UMIs per cell, and nCount_RNA represents the proportion of mitochondrial genes per cell.

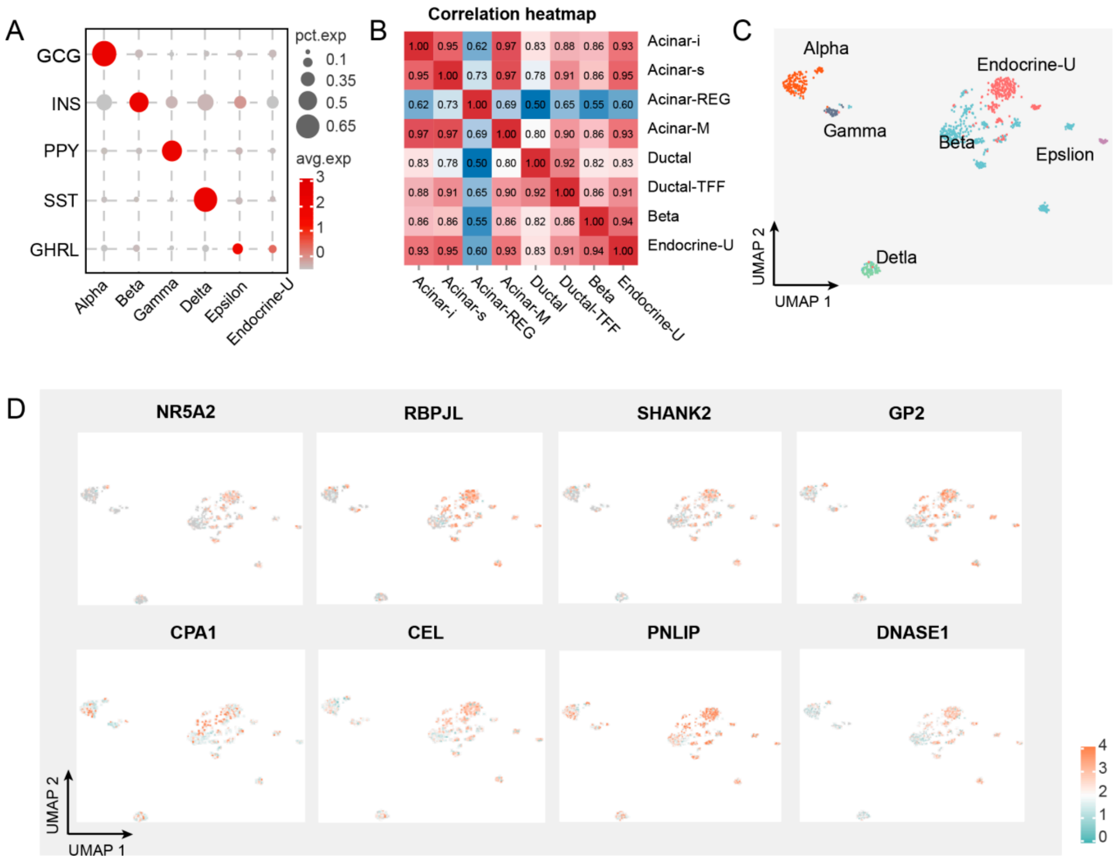

Supplementary Figure S2. Data related to Endocrine-U cluster. (**A**) Bubble plot of marker genes in endocrine cells. None of endocrine marker genes highly expression in the Endocrine-U cluster. (**B**) Correlation heatmap of each cell cluster (pearson). (**C**) The UMAP map of significantly increased genes in the Endocrine-U cluster.

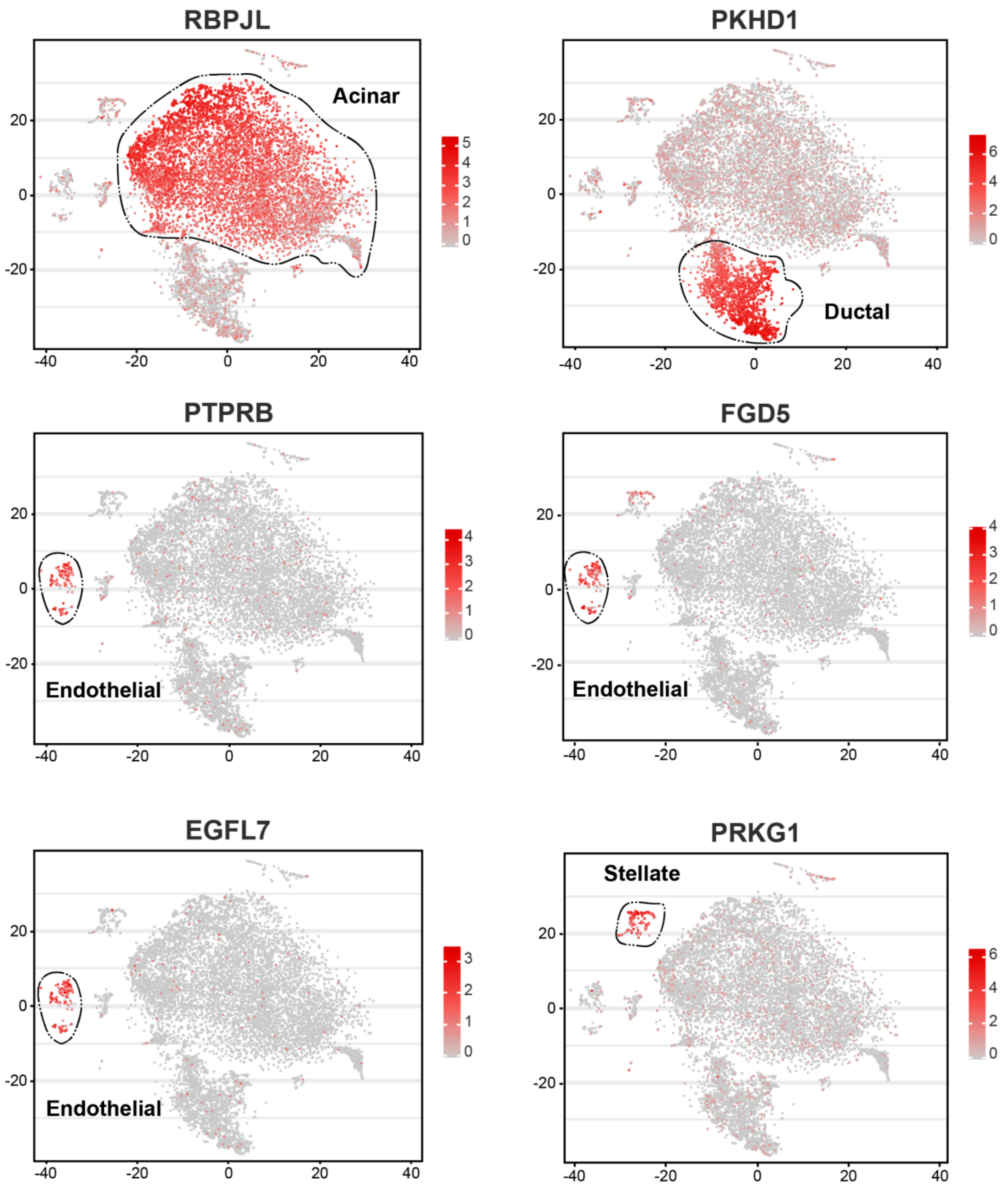

Supplementary Figure S3. The t-SNE map of potential marker gene in each cell type.

Supplementary Table S1. Data for quality control and genome alignment.

Group	WT	TG
Estimated Number of Cells	13,619	14,964
Number of Cells After Filtration	12,133	13,250
Fraction Reads in Cells	73.00%	71.30%
Mean Reads Per Cells	41,136	39,999
Median Genes Per Cells	1,651	818
Total Genes Detected	27,278	27,447
Median UMI Counts Per Cells	3,216	1,181
Reads Mapped Confidently to Genome	85.70%	82.50%
Reads Mapped Confidently to Integenic Regions	7.80%	10.00%
Reads Mapped Confidently to Intronic Regions	45.90%	50.20%
Reads Mapped Confidently to Exonic Regions	32.00%	22.30%
Reads Mapped Confidently to Transcriptome	58.80%	52.30%

Supplementary Table S2. Marker genes used in cell annotation.

Cell type	Genes
Acinar	PNLIP,CPA1,CPB1
Ductal	SCTR,CFTR,SCL4A4
Endothelial	CD36,VWF,FLT1
Stellate	PDGFRB,COL1A2
Macrophage	CD163,PTPRC
Schwann	CDH19,SCN7A
Alpha	GCG
Beta	INS
Gamma	PPY
Delta	SST
Epsilon	GHRL

Supplementary Table S3. Percentage of cell numbers of endocrine cells in pig

Cluster	WT	PIGinH11
**Alpha**	28.77%	12.31%
**Beta**	49.82%	40.06%
**Gamma**	2.46%	4.75%
**Delta**	9.82%	5.93%
**Epsilon**	0.70%	2.67%
**Endocrine-U**	8.42%	34.27%

Supplementary Table S4. Differential expression genes between TG and WT group in each cluster.

Cluster	Up-Genes	Down-Genes	Total-Genes
Acinar-i	103	168	271
Acinar-s	114	148	262
Acinar-REG	598	95	693
Acinar-M	156	298	454
Ductal	123	168	291
Ductal-TFF	248	203	451
Alpha	440	869	1309
Beta	513	1008	1521
Gamma	4	1024	1028
Delta	79	815	894
Endocrine-U	1032	2042	3074
Endothelial	304	371	675
Stellate	380	617	997
Macrophage	108	270	378
Schwann	215	465	680

Differential expression genes select criteria: $| \log FC | > 1$, $p < 0.05$.
